# Exceptionally Large Kidneys in Autosomal Dominant Polycystic Disease in India

**DOI:** 10.7759/cureus.13905

**Published:** 2021-03-15

**Authors:** Saher T Shiza, Jyothsna Guttikonda

**Affiliations:** 1 Internal Medicine, Deccan College of Medical Sciences, Hyderabad, IND; 2 Nephrology, Star Hospitals, Hyderabad, IND

**Keywords:** polycystic kidney, autosomal dominant, adpdk, nephrectomy

## Abstract

We report a case of exceptionally large kidneys in autosomal dominant polycystic kidney disease (ADPKD) in India. A 43-year-old male with a family history of ADPKD presented with abdominal pain, intermittent fever, and a sense of bilateral fullness in both flanks. On examination, he had bilaterally enlarged kidneys extending towards iliac fossae. The serum metabolic panel revealed elevated serum creatinine and blood urea nitrogen. Ultrasound abdomen and pelvis showed enlarged kidneys with parenchyma replaced by multiple varying-sized cysts and few cysts in both the kidneys leading to hemorrhagic transformation. CT abdomen showed bulky bilateral kidneys with multiple non-communicating cysts, with few cysts showing the hemorrhagic and calcific transformation. The right kidney measured 30.3 x 15 cm, weighing 9 lb, was resected. The left kidney measured 37.0 x 14.0 cm and was resected three months later. The specimen weighed 19.8 lb. Histopathological examination showed a gross specimen with a bossellated surface composed of sub-capsular multiple cysts of varying sizes. Both the enlarged kidneys were resected due to cyst hemorrhage and infection. The patient is currently on hemodialysis until he receives a renal graft.

## Introduction

Autosomal dominant polycystic kidney disease (ADPKD) affects all ethnic groups and is the most common monogenic kidney disease, with a prevalence of 1/500 to 1/1,000. It is also the commonest hereditary cause of the end-stage renal disease (ESRD). The development of multiple, bilateral fluid-filled renal cysts is a cardinal feature of ADPKD. Thousands of cysts of varying sizes typically develop in each kidney, often leading to massive kidney growth. Gross hematuria, proteinuria, hypertension, nephrolithiasis, and pain are also associated with larger kidney volumes leading to severe complications, including renal transplant [1]. Indications for surgical removal of an ADPKD kidney include intractable pain, hematuria, cyst infection, significantly enlarged kidneys, and small abdominal cavity hampering donor kidney implantation [2,3]. We report the case of bilaterally enlarged ADPKD kidneys of a 43-year-old male, with his largest kidney weighing 19.8 lb, with a maximal length of 37 cm, and with cysts filled with serous, hemorrhagic, and purulent material.

## Case presentation

A 43-year-old male with a family history of ADPKD presented to our hospital with severe abdominal pain and a sense of bilateral fullness in both flanks. He had intermittent pain in the lower abdomen radiating to the back and pelvis for the last three months, resolved by taking over counter analgesics. He also complained of intermittent low-grade fever and hematuria. On examination, he was thin built, averagely nourished with evident pallor. He had a temperature of 99^o^F, blood pressure of 155/105 mmHg, respiratory rate of 25 per minute. His abdomen was soft, non-tender, with bilateral enlarged kidney extending towards iliac fossae. His initial blood workup is shown in Table 1.

**Table 1:** Blood workup on admission WBC, white blood cell; RBC, red blood cell.

Parameter	Lab value	Reference range
WBC (cells per mm^3^)	15,000	4,000-11,000
RBC (million cells per mm^3^)	3.9	4.35-5.65
Platelet (cell per mm^3^)	140,000	150,000-350,000
Hemoglobin (mg/dL)	10.9	13.5-17

The serum metabolic panel was unremarkable except for elevated serum creatinine and blood urea nitrogen (Table 2). Urine analysis was positive for blood and albumin with no signs of infection.

**Table 2:** Serum metabolic panel on admission BUN, blood urea nitrogen; ALT, alanine aminotransferase; AST, aspartate aminotransferase; CRP, c-reactive protein; ESR, erythrocyte sedimentation rate.

Parameter	Lab value	Reference range
Serum creatinine (mg/dL)	10.9	0.7-1.2
BUN (mg/dL)	45	08-20
ALT (IU/L)	42	<42
AST (IU/L)	35	<45
CRP (mg/dL)	18	<10
ESR	30	<20
Prothrombin time (sec)	13	<13.5
Partial thromboplastin time (sec)	31	30-40
Sodium (mmol/L)	135	136-145
Potassium (mmol/L)	4.8	3.5-5.0
Chlorine (mmol/L)	99	98-106

**Figure 1:**
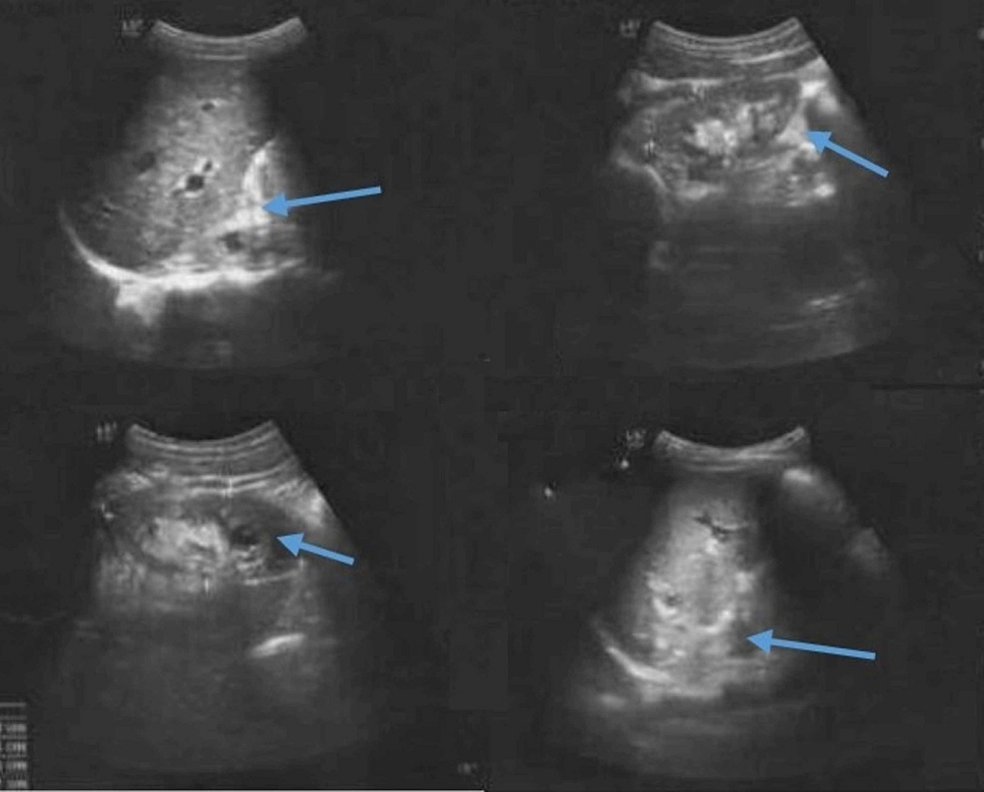
Ultrasound abdomen showing heterogeneous echotexture, cystic formation, and pulsation vascular center in the left kidney (blue arrows).

Ultrasound abdomen and pelvis showed enlarged kidneys with parenchyma replaced by multiple varying-sized cysts and few cysts in both the kidneys leading to hemorrhagic transformation (Figure 1). Mild hepatomegaly with parenchyma replaced by cysts was also seen.

**Figure 2:**
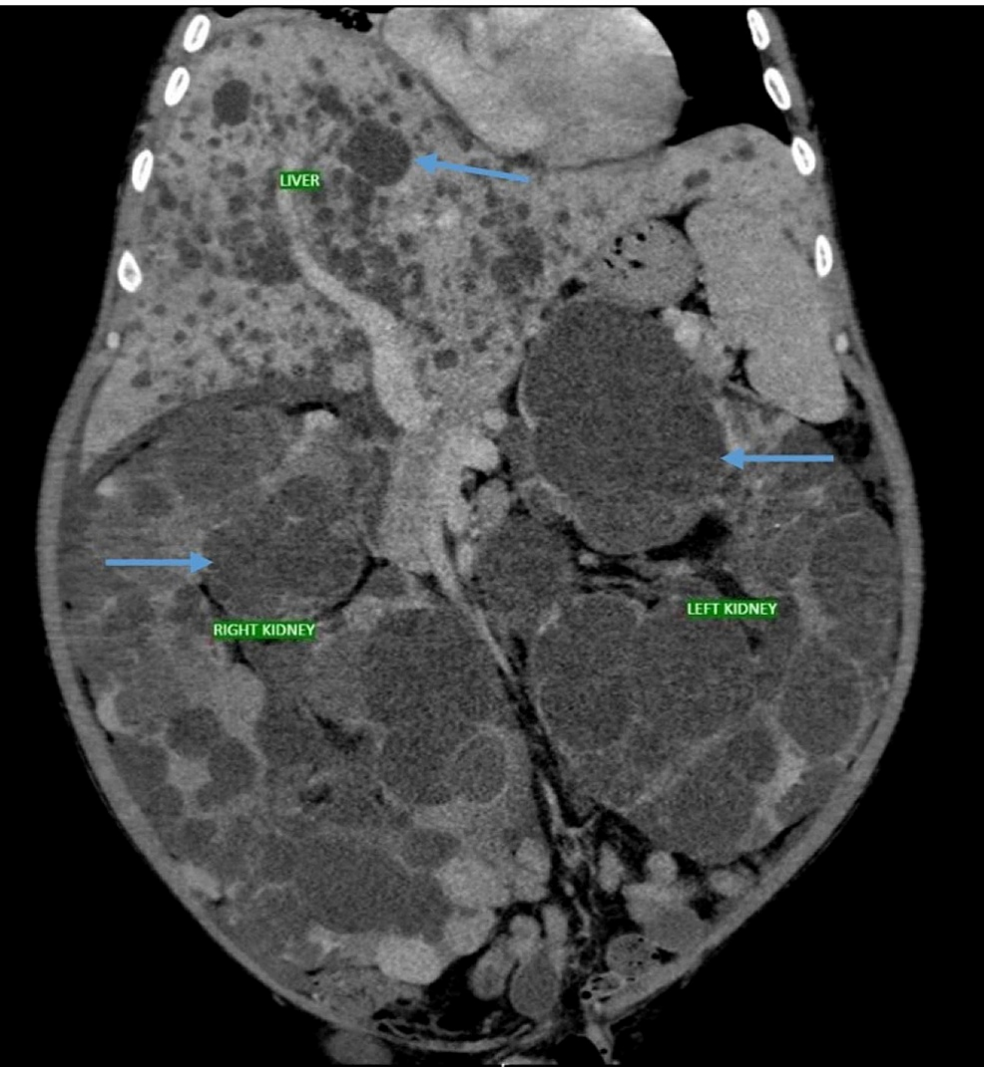
CT abdomen showing multiple varying-sized cysts replacing parenchyma in both kidneys and liver (blue arrows). CT; computed tomography.

Subsequent contrast-enhanced computed tomography (CT) abdomen showed bulky bilateral kidneys with multiple non-communicating cysts with few cysts showing the hemorrhagic and calcific transformation. The right kidney measured 30.3 x 15 cm and the left kidney measured 37.0 x 14.0 cm. MRI brain was also performed, which was unremarkable. Moderate hepatomegaly with varying-sized cysts replacing parenchyma and mild ascites was also noted (Figure 2). A right-sided nephrectomy was thus performed under general anesthesia.

**Figure 3:**
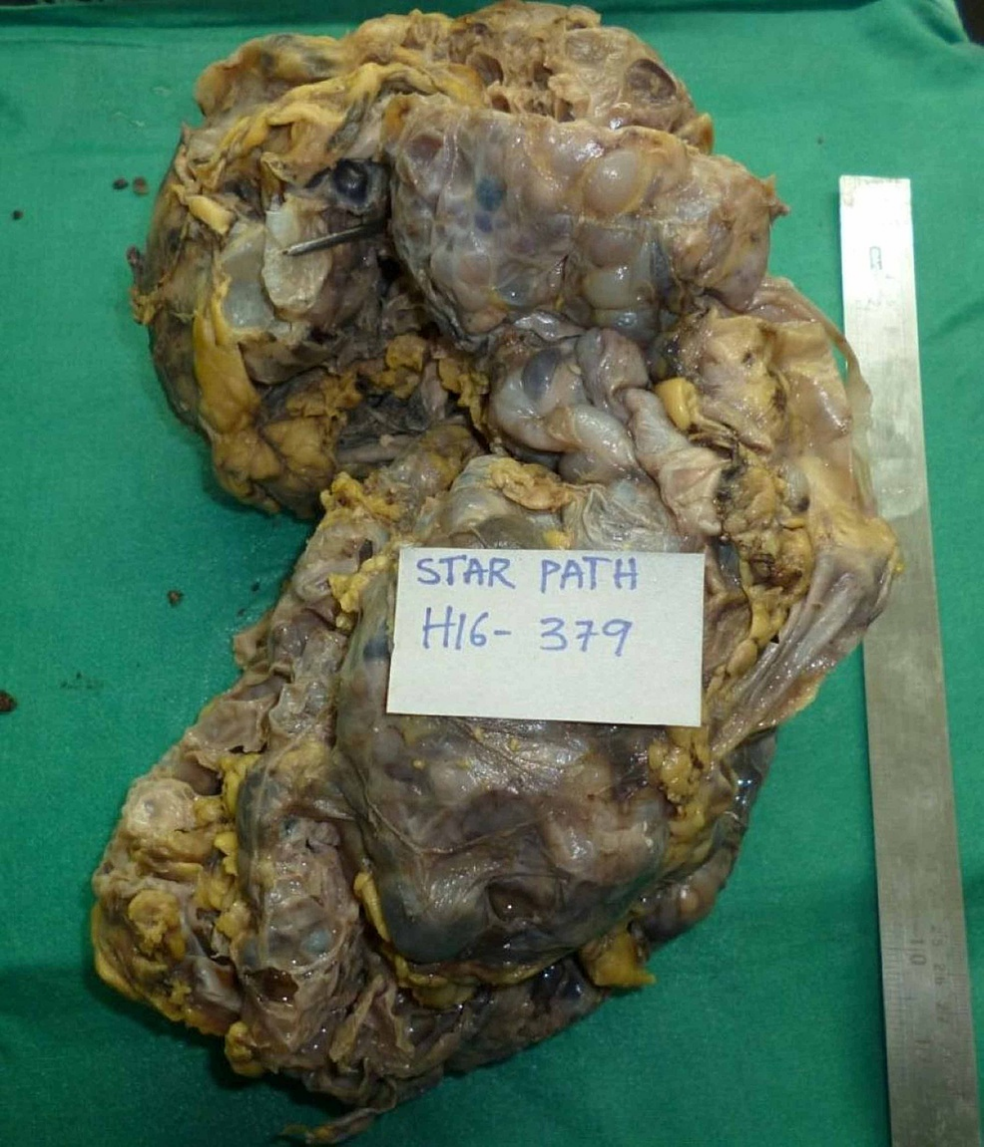
Resected left cystic kidney

Three months later, the patient presented again with complaints of hematuria associated with fever and chills. Lab values showed associated leukocytosis, anemia, and raised CRP. Examination showed grossly enlarged left palpable kidney causing severe abdominal discomfort and difficulty in breathing. Repeat ultrasound abdomen showed enlarged left kidney with multiple varying sized cysts replacing the parenchyma. Many of the cysts showed the presence of hemorrhagic changes. Serum creatinine was 11.14 mg/dL. Hence, the patient was planned for a left-sided nephrectomy. A massively enlarged left cystic kidney with its lower pole occupying the right iliac fossa was mobilized and resected. The specimen weighed 19.8 lb (Figure 3).

**Figure 4:**
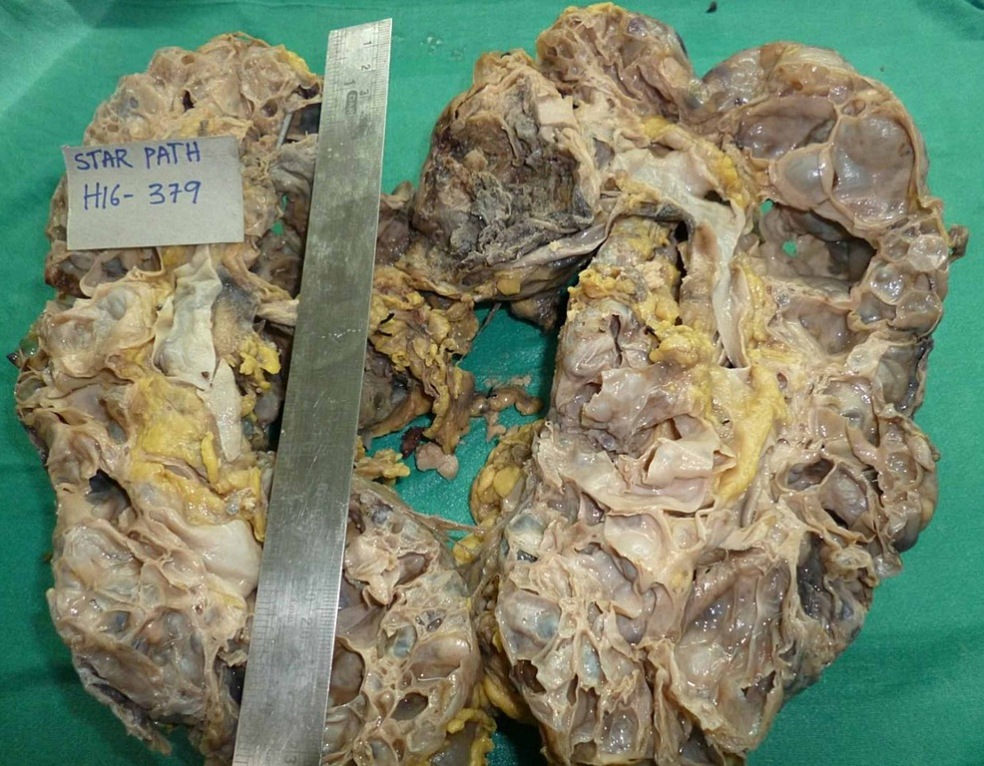
Cut section of the left kidney showing whole of the renal parenchyma replaced by cysts filled with serous, hemorrhagic, and necrotic material.

Histopathological examination showed a gross specimen measuring 37 x 20 x 10 cm with a bossellated surface composed of sub-capsular multiple cysts of varying sizes, ranging from 0.5 x 0.5 cm to 12 x 10 cm. The cut section showed cysts filled with serous, hemorrhagic, and purulent material. Few cysts also showed necrotic material. Two lymph nodes were identified at the hilar region measuring 2 x 1.5 x 1 cm and 2.8 x 1.5 x 1 cm (Figure 4). Microscopic examination showed renal parenchyma replaced by multiple cystic spaces of varying sizes. Some cysts were markedly dilated, lined with flattened cuboidal epithelium filled by eosinophilic proteinaceous material, foamy, and pigment-laden macrophages. There were foci of xanthogranulomatous changes with sheets of foamy macrophages, cholesterol clefts, giant cells, and collections of inflammatory cells. Insterstitium showed extensive hemorrhagic necrosis, neutrophilic cellular debris. Sections of the hilar lymph nodes showed reactive lymphoid hyperplasia. Both the enlarged kidneys were resected due to cyst hemorrhage and infection, and presently patient has been planned for renal replacement therapy with renal transplantation. The patient is currently on hemodialysis until he receives a renal graft.

## Discussion

To our knowledge, this is the first case in India reported with the given weight of the resected ADPKD kidney, with only two cases reported with ADPKD that had kidneys with higher total length and weight than the present case, outside India [2]. Polycystic kidney disease, being an autosomal dominant disorder, is a multisystem disease and progresses with kidney enlargement and cyst formation and the involvement of other body organs, including the liver, spleen, and pancreas.

ADPKD is the most common hereditary disease caused by mutations in PKD1 and PKD2 genes located on chromosomes 16p13.3 and 4q13q23. These mutations are usually present in more than 90% of the patients [4]. In adult patients, ADPKD is the most frequent cause of kidney failure (6%-8%). In our case, we did not perform the mutation analysis due to cysts in both kidneys and family history of ADPKD.

Clinical manifestation of ADPKD is related to the size of kidneys that increase with age and the severity of the structural abnormality. Kidney enlargement ultimately occurs in all the patients. Patients have a wide range of clinical manifestations, including pain, hematuria, vomiting, hypertension, and kidney dysfunction. In many cases, the clinical presentation is directly related to renal cyst enlargement [5]. Episodic renal colic is quite often due to cyst hemorrhage, infection, and stone. Visible hematuria can be the initial presentation of ADPKD due to cyst hemorrhage, which can present with fever, increasing the cyst infection possibility [6]. Urinary tract infections are common in APKD, and nephrolithiasis can also occur in ADPKD. Kidney morphology remains within the normal range in most patients. Kidneys get enlarged when the renal function starts worsening. At the age of 50, 77% of the patients are alive with normal kidney function, and 52% at 73. Untreated ADPKD can lead to ESRD, renal colic, infections, hypertension, polycystic liver disease, and cerebral aneurysms [7].

**Figure 5:**
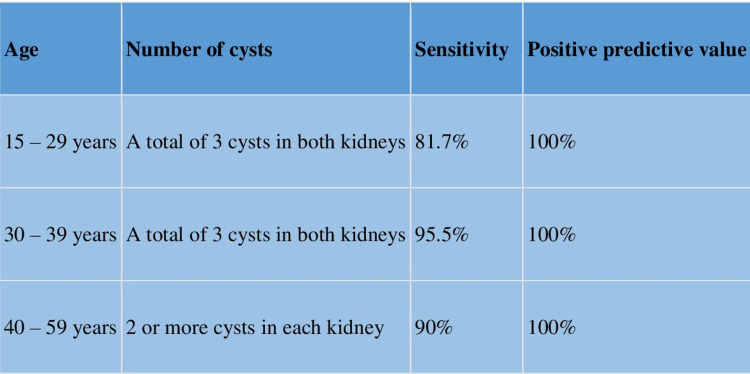
Criteria for ultrasound diagnosis of ADPKD ADPKD; autosomal dominant polycystic kidney disease.

The screening and diagnosis of ADPKD are based on clinical presentation, age, family history, and the number of cysts in patients. Manifestations include flank pain, early satiety, nausea, constipation, hematuria, and recurrent nephrolithiasis. Ultrasound being safe and cheaper is a useful modality for screening and diagnosis of ADPKD with higher chances. The unified criterion for the diagnosis of ADPKD is shown in figure 5 [8].

ADPKD can be managed symptomatically. Pain is usually managed with opioid analgesics in acute cases and tricyclic antidepressants in chronic cases [1]. Early management of cyst infection, hemorrhage, and UTI can improve the quality of life in ADPKD patients. Guidelines recommend proper control of blood pressure because uncontrolled hypertension in ADPKD speeds up the renal function decline. Drugs of choice for hypertension in ADPKD include angiotensin receptor blockers (ARB) or angiotensin-converting enzyme inhibitors (ACEIs) [1]. Lifestyle interventions that help delay the progression of ADPKD include limitation of dietary sodium, proper hydration, and healthy weight maintenance. The patients must be educated about the condition and its most likely complications. Tolvaptan is indicated in ADPKD to slow down the kidney growth and estimated glomerular filtration rate (GFR) decline in early disease. Therapeutic nephrectomy is advised for refractory and malignant ADPKD. 

## Conclusions

ADPKD is the most common hereditary kidney disease, has a significant impact on survival and quality of life. The comprehensive knowledge about the ADPKD, including its genetic heterogeneity and phenotype variation, is indispensable for accurate diagnosis and management. Early diagnosis and management are essential to prevent the complications of ADPKD. ADPKD can be managed with lifestyle medications, medications, and surgical intervention.
